# The Presence of *Treponema* spp. in Equine Hoof Canker Biopsies and Skin Samples from Bovine Digital Dermatitis Lesions

**DOI:** 10.3390/microorganisms9112190

**Published:** 2021-10-20

**Authors:** Paulína Marčeková, Marián Mad’ar, Eva Styková, Jana Kačírová, Miriam Sondorová, Pavol Mudroň, Zdeněk Žert

**Affiliations:** 1 Clinic of Ruminants, University Veterinary Hospital, University of Veterinary Medicine and Pharmacy in Košice, Komenského 73, 041 81 Košice, Slovakia; paulinamarcekova@gmail.com (P.M.); pavol.mudron@uvlf.sk (P.M.); 2 Department of Microbiology and Immunology, University of Veterinary Medicine and Pharmacy in Košice, Komenského 73, 041 81 Košice, Slovakia; kacirova.jana@gmail.com (J.K.); miriam.sondorova@gmail.com (M.S.); 3 Clinic of Horses, University Veterinary Hospital, University of Veterinary Medicine and Pharmacy in Košice, Komenského 73, 041 81 Košice, Slovakia; eva_stykova@yahoo.com (E.S.); zdenek.zert@uvlf.sk (Z.Ž.)

**Keywords:** equine hoof canker, bovine digital dermatitis, *Treponema*, PCR

## Abstract

Equine hoof canker and bovine digital dermatitis are infectious inflammatory diseases of the hooves with an unknown etiology. However, anaerobic spirochetes of the genus *Treponema* are considered to be potential etiological agents. The aim of this study was to find a suitable way to isolate DNA and to detect the presence of treponemal DNA in samples of equine hoof canker and bovine digital dermatitis. DNAzol^®®^ Direct and column kits were used to isolate DNA from samples of equine hoof canker and bovine digital dermatitis. The presence of *Treponema* spp. was detected using PCR and Sanger sequencing. DNAzol^®®^ Direct is suitable for isolating DNA from these types of samples. Treponemal DNA was detected in equine hoof samples as well as in bovine digital dermatitis skin samples. In equine hoof biopsies, the most frequently detected was *Treponema pedis* (8/13). *Treponema brennaborense* (2/13) and *Treponema denticola* (2/13) were also found. In the case of bovine digital dermatitis, *Treponema medium* ssp. *bovis* was confirmed in 14 of 36 skin samples. *Treponema pedis* (9/36), *Treponema vincentii* (1/36), *Treponema phagedenis* (1/36), and *Treponema brennaborense* (1/36) were detected as well. DNAzol^®®^ Direct was more appropriate for isolation of treponemal DNA because the columns isolation method was more equipment and time-consuming. The presence of several *Treponema* spp. was determined in the samples. In horses, the most commonly detected species was a *T. pedis*, while in cattle it was *T*. *medium* ssp. *bovis*.

## 1. Introduction

Bovine digital dermatitis (BDD) manifests as painful, ulcerative or proliferative lesions. Typical lesions have been classified based on clinical appearance into five stages [1] and are located mainly on the plantar aspect of the hind foot. This inflammatory disease of interdigital skin is one of the most common causes of lameness [2,3]. Pain and lameness disrupt animal welfare and have a negative impact on the economy of breeding such as a reduction in milk production [4,5].

The pathognomonic condition of equine hoof canker (*pododermatitis chronic verrucosa*) has a very similar clinical course, clinical signs and pathological appearance to BDD [6]. It endangers the use and welfare of horses due to the instability of the affected hoof capsule and the subsequent lameness [7]. This disease is described as an infectious process that is characterized by chronic, hypertrophic, moist subdermatitis which affects the horn-producing tissues. Hoof canker generally originates from the frog and associated sulcus region. It may remain in focus, but if left untreated, it can diffuse and invade the bars, adjacent sole, and even the hoof wall [8,9]. Hoof canker can occur in one or more hooves, but more often on the hindlimbs, although this is not common in horses. This disease can affect all breeds but working draft horses are affected more often [10,11].

*Treponema* spp., as a potential etiological agent, may play a role in the development and pathogenesis of both diseases [12,13]. This anaerobic or microaerophilic spirochetal bacterium can infect a wide range of hosts and tissues and cause a range of diseases from periodontal diseases in companion animals and humans [14,15] to digital dermatitis of cattle [16]. The shape and composition of the outer membrane, which has transmembrane proteins on its surface, probably contributes to the ability of bacteria to escape from the host’s immune system [17].

Several studies detected the presence of treponemal DNA in tissues from affected hooves, suggesting that treponemes may play an important role in the etiology and/or pathogenesis of diseases. [18,19,20]. The most common *Treponema* phylogroups associated with polytreponemal etiology of BDD are *Treponema medium*/*vincentii*-like, *Treponema phagedenis*, *Treponema denticola*-like/*putidum* and *Treponema pedis* [21,22,23], whereas *Treponema brennaborense* is rarely found in lesions [24]. The fragile and fastidious nature of genus *Treponema* makes their isolation and cultivation very difficult. Therefore, molecular approaches such as PCR are more commonly used to detect difficult-to-culture bacteria [25].

The aim of the present study was to find a suitable method for the isolation of treponemal DNA from equine hoof canker biopsies and BDD lesions, further standardization of PCR reactions to detect *Treponema* spp. and determine their presence in the samples.

## 2. Materials and Methods

### 2.1. Standardization of PCRs

PCR to detect *T. pedis*, *T. brennaborense* and group of *Treponema*, which include *T. denticola*, *T. vincentii*, *T. medium* ssp. *bovis*, and *T. phagedenis* ssp. *vaccae* were standardized. DNA samples, namely *T. denticola* DSM 14222, *T. brennaborense* DSM 12168, and *T. pedis* DSM 18691, obtained from the Deutsche Sammlung von Mikroorganismen und Zelikultren GmbH (DSMZ, Braunschweig, Germany) were used as positive controls. The primers designed in the study of Brandt et al. [20] and synthesized by Merck were used. The primers with modified PRC protocols are shown in Table 1. The products of the PCR reactions were purified and sequenced on both sides by the Sanger method (Microsynth, Vienna, Austria).

### 2.2. Collection of Equine Hoof Biopsies

Horses from two stud farms located in Slovakia and the Czech Republic were included in this study (Table 2). From five Czech warmblood mares, one Muran type norik mare and one Austrian norik stallion were collected hoof biopsies during debridement for histopathological examination, determination of the DNA presence of Treponema spp. and biopsy swabs for mycological examination. Hoof samples and swabs from one healthy Muran type norik stallion, one Austrian norik stallion and one Czech warmblood mare were taken as controls during regular trimming. Histopathological examination of hoof biopsies was provided by IDEXX laboratories (Leipzig, Germany) and mycological examination by the State Veterinary and Food Institute (Košice, Slovakia). Swabs for mycological examination were taken from the hoof biopsies and placed into the Amies agar gel without carbon (Copan, Mantua, Italy). To examine the presence of bovine papillomavirus 1 and 2 (BPV 1, 2), hoof tissue and hair with roots were taken from each horse from both the affected and healthy areas. The samples were sent to Laboklin s.r.o. (Bratislava, Slovakia).

### 2.3. Collection of Skin Samples from BDD Lesions

Samples were obtained from four farms in Eastern Slovakia (1—beef, 2, 3, 4—dairy). On the first farm breeding Charolais beef cattle, a sporadic occurrence of digital dermatitis was recorded at the time of sampling. Beef cattle spent the majority of the year on the pasture. Digital dermatitis on the second farm breeding dairy cattle had a high prevalence and was a long-term problem. The third farm was characterized by indoor breeding of dairy cattle and also the presence of digital dermatitis. The fourth farm had a loose housing system without pasture access and BDD is a permanent problem there. A more detailed description of the skin samples obtained from cattle patients with digital dermatitis is shown in Table 3.

### 2.4. Processing of Clinical Samples for Isolation and Detection of Treponemal DNA

Two different types of sample processing were used to isolate DNA from bioptic hoof samples. The first was dependent on isolation by DNAzol^®®^ Direct (Molecular Research Center Inc., Cincinnati, SA, USA) and the second one was based on Nucleo Spin Tissue columns (Macherey-Nagel GmbH & Co. KG, Düren, Germany). In the first case, shave biopsies of the hooves were taken from predilection sites using a scalpel. After collection, biopsies were placed in a 2 mL Eppendorf tube (Eppendorf, Hamburg, Germany) containing 100 μL DNAzol^®®^ Direct. The samples were then incubated for 15 min at 95 °C. The 1 μL DNA sample was used directly in the PCR reaction. In the second case of DNA isolation, the samples were frozen with liquid nitrogen immediately after collection and subsequently homogenized in stomacher bags using a mortar. DNA was isolated according to the manufacturer’s instructions Nucleo Spin Tissue (Macharey-Nagel GmbH & Co. KG). The same sample volume as DNAzol^®®^ Direct method was used in the PCR reaction. The One Taq^®®^ 2X Master Mix with Standard Buffer (New England Biolabs, Foster City, USA) in a final volume of 50 µL was used for the PCR reaction. Primers diluted to a concentration of 33 µM were used in a volume of 1 µL each. The DNA isolation process for bovine digital dermatitis was based on DNAzol^®®^ Direct and the PCRs were the same as used for the hoof canker biopsies. PCR products were visualized in 2% agarose gel electrophoresis under UV light and subsequently purified and sequenced on both sides by the Sanger method (Microsynth, Austria).

## 3. Results

**Figure 1:**
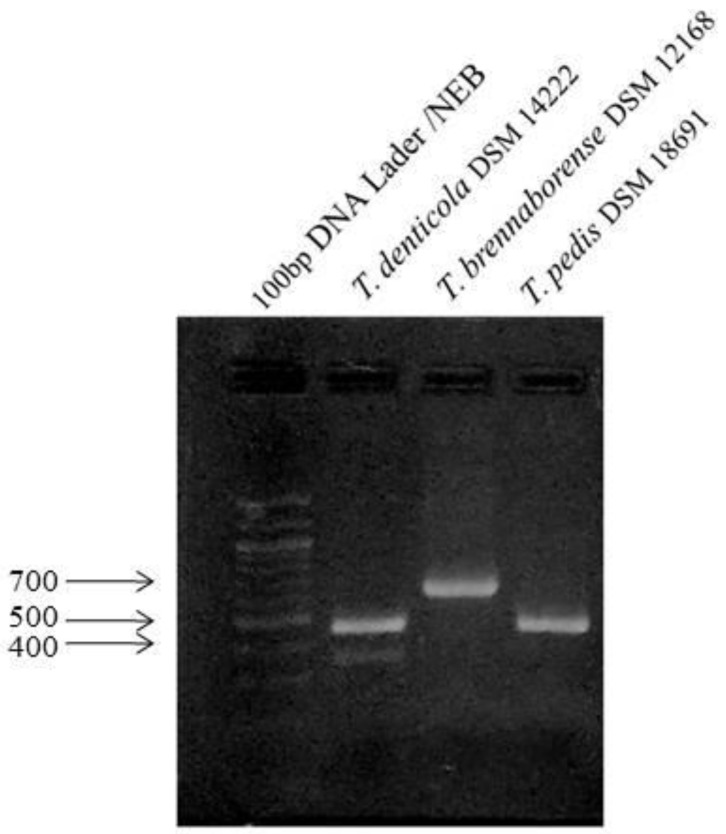
Detection of positive controls on agarose gel after amplification.

To accelerate the detection of treponemes in the samples, the annealing temperature was adjusted based on the melting temperature of primers. The same annealing temperature and the parameters of the PCR protocol suitable for the detection of *T. pedis* were also suitable for *T. brennaborense*. The presence of the products after amplification (Figure 1) and their subsequent sequencing verified the suitability of the PCR reaction even at the temperatures adjusted by us. Comparison of the obtained sequences with GenBank^®®^ DNA database using BLASTn analysis confirmed the sensitivity and specificity of these PCR reactions (Figures S1–S3).

### 3.1. Results of PCRs for Detection of Treponema spp., BPV (1, 2) Presence and Mycological Examination Results in Equine Hoof Biopsies

The clinically suspected diagnosis of *pododermatitis verrucosa chronica* (hoof canker), was confirmed by histopathological examination. The presence of treponemal DNA was confirmed in both stud farms (Table 4). *T. pedis* were detected in 8 out of 13 samples. *T. brennaborense* (2/13) and *T. denticola* (2/13) were also identified. *T. pedis* was found in horses with but also without hoof canker. All samples were negative for the presence of bovine papillomavirus (BPV 1, 2). Cultivation for dermatophytes on Sabouraud dextrose agar was negative in all samples. The mycological analysis revealed *Candida glabrata* (7/13), *Trichosporon* spp. (4/13), *Aspergillus terreus* (1/13), and *Penicillium* spp. (1/13).

### 3.2. Results of PCRs for Treponema spp. in Skin Samples from BDD Lesions

Representatives of *Treponema* spp. were detected in samples collected from skin lesions of cattle using specific PCRs. In cattle, *T. medium* ssp. *bovis* was detected in 14 samples from 36 samples (Table 5). *T. vincentii* (1/36) and *T. phagedenis* (1/36) were also identified. *T. pedis* was detected in nine samples and *T. brennaborense* in only one sample.

## 4. Discussion

Our results showed that DNA isolation for subsequent PCR using DNAzol^®®^ Direct was better for bioptic or skin samples, in contrast to the more time-consuming and necessary equipment (liquid nitrogen, centrifuge etc.) for DNA isolation with the Nucleospin tissue kit (Macharey-Nagel GmbH & Co. KG). During the collection and testing of clinical samples, it was found that the DNA isolation method using DNAzol^®®^ Direct is not suitable for long-term storage of DNA isolated from samples at 4 °C or −20 °C. Since the DNA is not purified and the samples remain in the DNAzol^®®^ Direct, which acts as a lysis solution, eventually the nature of the sample aids in their DNA degradation. Prior to processing with DNAzol^®®^ Direct, bioptic samples could be stored at −20 °C. Another limitation of the use of DNAzol is that it is not possible to measure the concentration of DNA obtained by spectrometry, as in the case of DNA extraction kits. However, this method was most suitable for rapid sample processing. According to Brandt et al. [20], we amplified a specific fragment of the 16S rRNA gene for the detection of *T. brennaborense* and the gene encoding flagellin *flaB2* for the detection of *T. pedis* and group *Treponema*, namely *T. denticola*, *T. vincentii*, *T. medium* ssp. *bovis,* and *T. phagedenis* spp. *vaccae*. The primers used to detect the group *Treponema* encode a variable region, and sequencing is necessary to identify treponemal species. While PCR for detection *T. brennaborense* and *T. pedis* is species-specific.

Equine hoof canker is usually associated with unhygienic conditions. However, it has also been reported in horses with well-maintained hygienic conditions [6]. Microorganisms such as fungi, anaerobic bacteria, viruses, especially bovine papillomavirus, as well as genetic predisposition and autoimmune reactions are involved in the development of this disease [7]. The presence of *Treponema* spp. has been determined not only in equine hoof canker but also in horses without hoof canker. Sequences of DNA identical or similar to bovine digital dermatitis associated with *T. medium* ssp. *bovis* and *T. denticola* were exclusively detected in equine hoof canker samples′, while *Treponema refringens*, *T. pedis,* and *T. phagedenis* were detected in horses with healthy hooves [12]. In this study, the presence of the treponemal DNA was detected in samples of hooves with and without equine hoof canker, which is consistent with previous findings. Moe et al. [19] reported that treponemal DNA is not only present in hooves affected with equine hoof canker. Bacteria of genus *Treponema* are a physiological part of the intestinal microbiota of horses, with most commonly present *Treponema bryantii and Treponema succinifaciens* [26,27]. The most detected species of *Treponema* in our tested samples from horses was *T. pedis*, while *T. brennaborense* DNA and *T. denticola* DNA were detected, but in a few cases. The presence of *T. brennaborense* and *T. denticola* was also reported in a previous study using clonal sequence analysis of 16S rRNA [19]. Brandt et al. [11], revealed the presence of BPV 1 and 2 in all samples with equine hoof canker, whereas there were no BPV 1 and 2 detected in our samples. Although BDD is considered to be a multifactorial polymicrobial disease, *Treponema* spp. are the most common bacteria associated with this disease [28]. Detection of these difficult-to-cultivate bacteria by PCR is considered a rapid and sensitive diagnostic method. In the study by Brandt et al. [20], *Treponema* spp. were detected in 38 samples from 45 samples by quantitative PCR. Qualitative PCR revealed *Treponema* spp. in 42 samples from 45 samples. *T. pedis* was the most frequently detected treponemal species found in 51% of the samples, while *T. brennaborense* was not found in any of the samples in their study. In the present study, the most common species was *T. medium* ssp. *bovis* detected in 39% of the samples. We detected only one positive sample for *T. brennaborense*, which is reported as less frequent also by Wilson and Welder [29]. In the study by Mamuad et al. [16], samples were positively tested for *T. phagedenis*-like (86.2%), *T. medium*/*T. vincentii*-like (75%), and *T. pedis* (68.8%). In 25% of the lesions all the phylogroups were detected and phylogroup *T. putidum*/*T. denticola*-like was the most frequent in the lesions. Nascimento et al. [25] detected all phylogroups in 81.8% of lesions. Bomjardin et al. [23] explain that lower frequency with low diversity of *Treponema* found in the lesions may be related to the lower environmental pressure to these animals raised on pasture compared to intensively reared dairy cows from the study of Nacimiento et al. [25]. Moreira et al. [30] reported a lower prevalence (72.9%) of treponemal species in BDD lesions from dairy cows in different regions of Brazil. This difference indicates the influence of the breeding environment on the frequency of treponemes. In the Alsaaod et al. [31] study, the most prevalent was *T. phagedenis* (65.1%). Beninger et al. [32] reported significantly higher amounts of *Treponema* spp. in the active ulcerative lesions than in healing and chronic ones. Bomjadrim et al. [23] and Moreira et al. [30] suggest the influence of the stages of BDD lesions on the possibility to detect etiological agents. Their results suggest that BDD lesions in the early stages of the disease and in the healing stage may have a low concentration of bacteria in the affected tissues or may even lack any presence of Treponema. Treponemes can also be localized in a non-homogenous manner in which biopsies are not able to obtain proper genetic material for diagnosis. Above mentioned reasons and long-term storage of samples in −20 °C could be a possible reason for the low number of positive results in our study compared to other referred.

## 5. Conclusions

DNA isolation with DNAzol^®®^ Direct, PCR, Sanger sequencing, and genotyping are suitable for finding the etiological agents of equine hoof canker and bovine digital dermatitis. In the present study, several species of *Treponema* have been detected in samples of equine hoof canker and bovine digital dermatitis. *T. pedis* was the most commonly detected species in horses. *T. medium* ssp. *bovis* and *T. pedis* were frequent in cattle. Future analysis of either BDD or equine hoof canker could focus on other types of samples that could be a potential source of *Treponema* spp. such as fecal samples from the rectum, fecal samples over the lesion, various skin layers, and others.

## Figures and Tables

**Table 1:** PCR conditions for detection of *Treponema* spp.

Detected Species (Gene)	Primers	PCR Protocol	Product
*Treponema pedis* (*flaB2*)	TPed32f: 5′-CTTACTTACAGGAAACTACGGAC-3′; Tped-500r: 5′-GCAATGTTAATTCCTACAACCGTAAG-3′	94 °C 5min 35x (94 °C 30 sec, 61 °C 30 s,72 °C 40 sec) 72 °C 5 min	424 bp
*Treponema brennaborense* (16SrRNA)	TBrenn-418f: 5′-GACAGCGTGGTGACAGTAGG-3′; TBrenn-1080r: 5′-CTTGCTGGTAACTGGCAGTAGG-3′	94 °C 5 min 35x (94 °C 30 s, 61 °C 30 s,72 °C 40 s) 72 °C 5 min	663 bp
*Treponema denticola*, *Treponema vincentii*, *Treponema medium* ssp. *bovis*, *Treponema phagedenis* spp. *vaccae* (*flaB2*)	TMult-2f: 5′-ACGGYATTTCYTTTATTCAAGTTGC-3′; TMult-472r: 5′-CGAGTCTGTTYTGGTATGCACC-3′	94 °C 5 min, 45x (94 °C 30 s, 63 °C 30 s, 72 °C 40 s) 72 °C 5 min	471 bp

**Table 2:** Specification of equine hoof biopsies samples.

Horse	Age (years)	Sex	Breed	Diagnosis	Affected Leg	Location
1.1	14	♀	CW	canker	RH frog	Pardubice region
1.2	14	♀	CW	canker	LH frog	Pardubice region
2	12	♀	CW	canker	LH frog	Pardubice region
3.1	20	♀	CW	canker	RH frog	Pardubice region
3.2	20	♀	CW	canker	LH frog	Pardubice region
4.1	13	♀	CW	canker	RH heel	Pardubice region
4.2	13	♀	CW	canker	LH heel	Pardubice region
5	19	♀	CW	canker	LH frog	Pardubice region
6	5	♀	CW	healthy	LF frog	Pardubice region
7	16	♀	MTN	canker	RF frog	Košice region
8	9	♂	AN	canker	RF frog	Košice region
9	12	♂	AN	healthy	LH frog	Košice region
10	8	♂	MTN	healthy	RF frog	Košice region

CW: Czech warmblood; AN: Austrian norik; MTN: Muran type norik; RF: right foreleg; LF: left foreleg; LH: left hindleg.

**Table 3:** Specification of skin samples in cattle with digital dermatitis.

Cow	Age (years)	Sex	Breed	Farm
1	3	♀	Ch	1
2	2	♀	Ch	1
3	4	♀	Ch	1
4	4	♀	Ch	1
5	3	♀	Ch	1
6	2	♀	HF	2
7	3	♀	HF	2
8	3	♀	HF	2
9	7	♀	HF	2
10	2	♀	HF	2
11	2	♀	S	2
12	3	♀	S	2
13	6	♀	HF	2
14	7	♀	HF	2
15	3	♀	HF	2
16	4	♀	HF	2
17	3	♀	HF	2
18	5	♀	HF	2
19	2	♀	HF	2
20	3	♀	HF	2
21	5	♀	HF	2
22	7	♀	S	3
23	4	♀	S	3
24	7	♀	S	3
25	3	♀	HF	2
26	5	♀	HF	2
27	3	♀	HF	2
28	3	♀	HF	2
29	5	♀	HF	2
30	4	♀	HF	2
31	4	♀	S	4
32	5	♀	S	4
33	3	♀	S	4
34	6	♀	S	4
35	3	♀	S	4
36	4	♀	S	4

Ch: Charolais; HF: Holstein Friesian; S: Siemental.

**Table 4:** *Treponema* spp. detection and mycological examination results of equine hoof biopsies.

Horse	Diagnosis	Affected Leg	Closest Related Sequence (BLASTn Homology)	Identity (%)	Mycological Examination
1.1	canker	RH frog	*Treponema pedis* *Treponema brennaborense*	100 99	*Trichosporon* spp. *Candida glabrata*
1.2	canker	LH frog	*Treponema pedis*	100	*Candida glabrata*
2.	canker	LH frog	*Treponema pedis* *Treponema denticola*	100 98	*Candida glabrata*
3.1	canker	RH frog	*Treponema pedis* *Treponema brennaborense* *Treponema denticola*	100 98 99	*Trichosporon* spp. *Aspergillus terreus*
3.2	canker	LH frog	*Treponema pedis*	100	*Candida glabrata* *Trichosporon* spp.
4.1	canker	RH heel	negative		*Candida glabrata*
4.2	canker	LH heel	*Treponema pedis*	100	*Candida glabrata*
5	canker	LH frog	negative		*Trichosporon* spp. *Penicillium* spp.
6	healthy	LF frog	*Treponema pedis*	100	*Candida glabrata*
7	canker	RF frog	negative		ND
8.	canker	RF frog	*Treponema pedis*	100	ND
9	healthy	LH frog	negative		ND
10	healthy	RF frog	negative		ND

RF: right foreleg; LF: left foreleg; LH: left hindleg; ND: not detected.

**Table 5:** *Treponema* spp. detection of samples from BDD lesions.

Cow	Closest Related Sequence (BLASTn Homology)	Identity (%)
1	*Treponema medium* ssp. *bovis*	98
2	*Treponema pedis*	100
3	*Treponema medium* ssp. *bovis*	98
4	*Treponema pedis* *Treponema brennaborense*	100 100
5	*Treponema pedisTreponema medium* ssp. *bovis*	100 98
6	negative	
7	*Treponema medium* ssp. *bovis*	98
8	negative	
9	negative	
10	negative	
11	*Treponema medium* ssp. *bovis*	100
12	*Treponema medium* ssp. *bovis*	100
13	*Treponema medium* ssp. *bovis*	99
14	negative	
15	*Treponema medium* ssp. *bovis*	100
16	*Treponema medium* ssp. *bovis*	97
17	negative	
18	negative	
19	negative	
20	negative	
21	*Treponema medium* ssp. *bovis*	99
22	negative	
23	*Treponema pedis*	100
24	negative	
25	*Treponema pedisTreponema medium* ssp. *bovis*	100 100
26	*Treponema medium* ssp. *bovis*	99
27	*Treponema vincentii*	89
28	*Treponema pedis* *Treponema phagedenis*	100 95
29	*Treponema pedisTreponema medium* ssp. *bovis*	100 99
30	*Treponema pedisTreponema medium* ssp. *bovis*	100 100
31	negative	
32	negative	
33	negative	
34	negative	
35	negative	
36	*Treponema pedis*	100

## Data Availability

The data presented in this study are available on request from the corresponding author.

## Supplementary Materials

The following are available online at www.mdpi.com/article/10.3390/microorganisms9112190/s1, Figure S1: The consensus sequence of flaB2 gene obtained after sequencing and assembling Treponema pedis DSM 18691 as a positive control, Figure S2: The consensus sequence of flaB2 gene obtained after sequencing and assembling Treponema denticola DSM 14222 as a positive control, Figure S3: The consensus sequence of 16S rRNA gene obtained after sequencing and assembling *Treponema brennaborense* DSM 12168 as a positive control.

## References

[1] Gillespie A.V., Carter S.D., Blowey R.W., Staton G.J., Evans N.J. (2020). Removal of bovine digital dermatitis-associated treponemes from hoof knives after foot-trimming: A disinfection field study. BMC Vet. Res..

[2] Staton G.J., Sullivan L.E., Blowey R.W., Carter S.D., Evans N.J. (2020). Surveying bovine digital dermatitis and non-healing bovine foot lesions for the presence of *Fusobacterium necrophorum*, *Porphyromonas endodontalis* and *Treponema pallidum*. Vet Rec..

[3] Newbrook K., Carter S.D., Crosby-Durrani H., Evans N.J. (2021). Challenge of Bovine Foot Skin Fibroblasts with Digital Dermatitis Treponemes Identifies Distinct Pathogenic Mechanisms. Front. Cell. Infect. Microbiol..

[4] Hartshorn R.E., Thomas E.C., Anklam K., Lopez-Benavides M.G., Buchalova M., Hemling T.C., Döpfer D. (2013). Short communication: Minimum bactericidal concentration of disinfectants evaluated for bovine digital dermatitis-associated *Treponema phagedenis*-like spirochetes. J. Dairy Sci..

[5] Anklam K., Kulow M., Yamazaki W., Döpfer D. (2017). Development of real-time PCR and loop-mediated isothermal amplification (LAMP) assays for the differential detection of digital dermatitis associated treponemes. PLoS ONE.

[6] Kuwano A., Niwa H., Higuchi T., Mitsui H., Agne R.A. (2012). Treponemes-infected canker in a Japanese racehorse: Efficacy of maggot debridement therapy. J. Equine Sci..

[7] Apprich V., Licka T., Zipfl N., Tichy A., Gabriel C. (2017). Equine Hoof Canker: Cell Proliferation and Morphology. Vet. Pathol..

[8] Oosterlinck M., Deneut K., Dumoulin M., Gasthuys F., Pille F. (2011). Retrospective study on 30 horses with chronic proliferative pododermatitis (canker). Equine Vet. Educ..

[9] Azzolini E.F.O.T., Bastos S.F., Barros R.M. (2019). Chronic equine proliferative pododermatitis: Case report *Braz*. J. Vet. Res. Anim. Sci..

[10] Fürst A.E., Lische C.J., Auer J.A., Stick J.A. (2012). Chapter 90-Foot. Equine Surgery.

[11] Brandt S., Schoster A., Tober R., Kainzbauer C., Burgstaller J.P., Haralambus R., Steinborn R., Hinterhofer C., Stanek C. (2011). Consistent detection of bovine papillomavirus in lesions, intact skin and peripheral blood mononuclear cells of horses affected by hoof canker. Equine Vet. J..

[12] Sykora S., Brandt S. (2015). Occurrence of *Treponema* DNA in equine hoof canker and normal hoof tissue. Equine Vet. J..

[13] Espiritu H.M., Mamuad L.L., Jin S.J., Kim S.H., Kwon S.W., Lee S.S., Lee S.M., Cho Y.I. (2020). Genotypic and Phenotypic Characterization of *Treponema phagedenis* from Bovine Digital Dermatitis. Microorganisms.

[14] Nises J., Rosander A., Pettersson A., Backhans A. (2018). The occurrence of *Treponema* spp. in gingival plaque from dogs with varying degree of periodontal disease. PLoS ONE..

[15] Tanno-Nakanishi M., Kikuchi Y., Kokubu E., Yamada S., Ishihara K. (2018). *Treponema denticola* transcriptional profiles in serum-restricted conditions. FEMS Microbiol. Lett..

[16] Mamuad L.L., Seo B.J., Faruk M.S.A., Espiritu H.M., Jin S.J., Kim W.I., Lee S.S., Cho Y.I. (2020). *Treponema* spp., the dominant pathogen in the lesion of bovine digital dermatitis and its characterization in dairy cattle. Vet. Microbiol..

[17] Buyuktimkin B., Zafar H., Saier M.H. (2019). Comparative genomics of the transportome of Ten *Treponema* species. Microb. Pathog..

[18] Nagamine C.M., Castro F., Buchanan B., Schumacher J., Craig J.L.E. (2005). Proliferative pododermatitis (canker) with intralesional spirochetes in three horses. Vet. Diagn. Investig..

[19] Moe K.K., Yano T., Kuwano A., Sasaki S., Misawa N. (2010). Detection of treponemes in canker lesions of horses by 16S rRNA clonal sequencing analysis. J. Vet. Med. Sci..

[20] Brandt S., Apprich V., Hackl V., Tober R., Danzer M., Kainzbauer C., Gabriel C., Stanek C., Kofler J. (2011). Prevalence of bovine papillomavirus and *Treponema* DNA in bovine digital dermatitis lesions. Vet. Microbiol..

[21] Klitgaard K., Nielsen M.W., Ingerslev H.C., Boye M., Jensen T.K. (2014). Discovery of bovine digital dermatitis-associated *Treponema* spp. in the dairy herd environment by a targeted deep-sequencing approach. Appl. Environ. Microbiol..

[22] Kuhnert P., Brodard I., Alsaaod M., Steiner A., Stoffel M.H., Jores J. (2020). *Treponema phagedenis* (ex Noguchi 1912) Brumpt 1922 sp. nov., nom. rev., isolated from bovine digital dermatitis. Int. J. Syst. Evol. Microbiol..

[23] Bomjardim H.A., Oliveira M.C., Cordeiro M.D., Brito M.F., Fonseca A.H., Oliveira C., Silva N.S., Barbosa J.D. (2020). Detection of *Treponema* spp. in bovine digital dermatitis in the Amazon biome, Brazil. Pesquisa Veterinária Brasileira.

[24] Nordhoff M., Moter A., Schrank K., Wieler L.H. (2008). High prevalence of treponemes in bovine digital dermatitis-a molecular epidemiology. Vet. Microbiol..

[25] Nascimento L.V., Mauerwerk M.T., Dos Santos C.L., Barros Filho I.R., BirgelJúnior E.H., Sotomaior C.S., Madeira H.M., Ollhoff R.D. (2015). Treponemes detected in digital dermatitis lesions in Brazilian dairy cattle and possible host reservoirs of infection. J. Clin. Microbiol..

[26] Daly K., Stewart C.S., Flint H.J., Shirazi-Beechey S.P. (2001). Bacterial diversity within the equine large intestine as revealed by molecular analysis of cloned 16S rRNA genes. FEMS Microbiol. Ecol..

[27] Steelman S.M., Chowdhary B.P., Dowd S., Suchodolski J., Janečka J.E. (2012). Pyrosequencing of 16S rRNA genes in fecal samples reveals high diversity of hindgut microflora in horses and potential links to chronic laminitis. BMC Vet. Res..

[28] Demirkan I., Erdoğan M., Demirkan A.Ç., Bozkurt F., Altındiş M., Navruz F.Z., Köse Z. (2018). Isolation and identification of *Treponema pedis* and *Treponema phagedenis*-like organisms from bovine digital dermatitis lesions found in dairy cattle in Turkey. J. Dairy Sci..

[29] Wilson-Welder J.H., Alt D.P., Nally J.E. (2015). Digital Dermatitis in Cattle: Current Bacterial and Immunological Findings. Animals.

[30] Moreira T.F., Facury Filho E.J., Carvalho A.U., Strube M.L., Nielsen M.W., Klitgaard K., Jensen T.K. (2018). Pathology and bacteria related to digital dermatitis in dairy cattle in all year round grazing system in Brazil. PLoS ONE.

[31] Alsaaod M., Locher I., Jores J., Grimm P., Brodard I., Steiner A., Kuhnert P. (2019). Detection of specific *Treponema* species and *Dichelobacter nodosus*from digital dermatitis (Mortellaro’s disease) lesions in Swiss cattle. Schweiz. Arch. Tierheilkd..

[32] Beninger C., Naqvi S.A., Naushad S., Orsel K., Luby C., Derakhshani H., Khafipour E., De Buck J. (2018). Associations between digital dermatitis lesion grades in dairy cattle and the quantities of four *Treponema* species. Vet. Res..

