# Supplementary material for: The Presence of Treponema spp. in Equine Hoof Canker Biopsies and Skin Samples from Bovine Digital Dermatitis Lesions
Source: Microorganisms. 2021 Oct 20;9(11):2190. doi: 10.3390/microorganisms9112190 (PMC8625648; doi:10.3390/microorganisms9112190)
Supplement: Supplementary file 1 [file microorganisms-09-02190-s001.zip › Supplementary material Figure S1-S3.pdf]

AAACTACGGACATTATGCAGAGAATCAGGGAGTTGGCCGTTCAAGCTTCTAACGGTAT
TTATTCCGCAGAAGACAGAATGCAAATTCAAGTTGAAGTTTCTCAGCTGGTTGCTGAA
GTTGACCGCATTGCAAGTTCCGCACAATTTAACGGAATGAATATGCTTACGGGCCGCT
TTGCACGTGAGACCGGTGAAAACGTTGTTACCGCTTCCATGTGGTTCCACATCGGTGC
AAACATGGACCAAAGAATGCGCGTTTACATCGGAACAATGTCGGCTGCAGCCGTAGG
AATTCGCGAAATCGGTTCGGAAAAGATTATGACAATCGAAACTGCCGATTCCGCCAAT
ATGAGCATCGGAACGATTGATGAAGGCTTAAAGAAAATCAATAAGCAAAGAGCGGAC
CTCGGAGCTTACCAGAACAGAATGGAACTTACGGTTGTAGGA

Figure S1 The consensus sequence of *flaB2* gene obtained after sequencing and assembling *Treponema pedis* DSM 18691 as a positive control.

TTTTATTCAAGTTGCGGAAGCTTTCCTTCAGGAAACTACAGATGTTATCCAGAGAATCC
GCGAACTCAGCATTCAGTCTTCTAACGGTATTTACTCGGCAGAAGACAGATTGTACATT
CAGGTTGAAGTATCTCAGCTAATCGCTGAAGTAGACCGAATTGCAAGCCATGCACAGT
TCAACGGTATGAATATGCTTACCGGAAGATTTGCTCAAGAAACCGGAGAAAATACCGT
AACTGCTTCTATGTGGTTCCACATCGGTGCCAACATGGATCAGAGAACAAGAGCTTAC
ATTGGAACAATGACAGCTAAGGCTCTCGGCGTTCGCAATATCGGAGATGAATCGATTA
TGACTATCGAAACACCCGAAAAAGCTAACCGCGCTATCGGTACCCTTGATGAAGCCAT
CAAGAAGATCAACAAGCAAAGAGCCGACCTTGGTGCATACCAG

Figure S2 The consensus sequence of *flaB2* gene obtained after sequencing and assembling *Treponema denticola* DSM 14222 as a positive control.

CTGGCAGTAGGGGTTGCGCTCGTTGCGGGACTTAACCCAACACCTCACGGCACGAGC

TGACGACAGCCATGCAGCACCTGTATACCGGCGTATTGCTACGCTCTGTTGTCTCCAAC

ACATTCCAGTATATGTCAAACCCAGGTAAGGTTCCTCGCGTACCATCGAATTAAACCAC

ATGCTCCACCGCTTGTGCGGGCCCCCGTCAATTCCTTTGAGTTTCACCCTTGCGGGCAT

ACTTCCCAGGCGGTACACTTATCACGTTTGCTTTGGCACCCAGTCTCTTGACCAGACA

CCTAGTGTACATCGTTTACTGTGCGGACTACCAGGGTATCTAATCCTGTTCGCTCCCCG

CACCTTCGCACCTCAGCGTCAGTTATCTGCCTGAAACTTGCCTTCGCCATTGGTGTTCT

TCCAGATATCTACAGATTTCACCCCTACACCTGGAATTCCAGTTTCACTTCAGTAACTCT

AGTCTTATAGTTCTCAATGCGGTTCCGGGGTTGAGCCCCGGGATTTCACACCAAGCTT

GCAAAACTGCCTACATGCCCTTTACGCCCAATAATTCCGAACAACGCTCGCAACTTACG

TGTTACCGCGGCTGCTGGCACGTAATTAGCCGTTGCTTATTCAAAACCTACT

Figure S3 The consensus sequence of 16S rRNA gene obtained after sequencing and assembling *Treponema brennaborense* DSM 12168 as a positive control.
